# Enhancement of Sphingolipid Synthesis Improves Osmotic Tolerance of Saccharomyces cerevisiae

**DOI:** 10.1128/AEM.02911-19

**Published:** 2020-04-01

**Authors:** Guoxing Zhu, Nannan Yin, Qiuling Luo, Jia Liu, Xiulai Chen, Liming Liu, Jianrong Wu

**Affiliations:** a State Key Laboratory of Food Science and Technology, Jiangnan University, Wuxi, China; b Key Laboratory of Industrial Biotechnology, Ministry of Education, Jiangnan University, Wuxi, China; c Key Laboratory of Carbohydrate Chemistry and Biotechnology, Ministry of Education, School of Biotechnology, Jiangnan University, Wuxi, China; Shanghai Jiao Tong University

**Keywords:** adaptive laboratory evolution, complex sphingolipid, membrane engineering, membrane integrity, osmotic tolerance

## Abstract

To enhance the growth performance of Saccharomyces cerevisiae under osmotic stress, mutant XCG001, which tolerates up to 1.5 M NaCl, was isolated through adaptive laboratory evolution (ALE). Comparisons of the transcriptome data of mutant XCG001 and the wild-type strain identified *ELO2* as being associated with osmotic tolerance. In the *ELO2* overexpression strain (XCG010), the contents of inositol phosphorylceramide (IPC; t18:0/26:0), mannosylinositol phosphorylceramide [MIPC; t18:0/22:0(2OH)], MIPC (d18:0/22:0), MIPC (d20:0/24:0), mannosyldiinositol phosphorylceramide [M(IP)_2_C; d20:0/26:0], M(IP)_2_C [t18:0/26:0(2OH)], and M(IP)_2_C [d20:0/26:0(2OH)] increased by 88.3 times, 167 times, 63.3 times, 23.9 times, 27.9 times, 114 times, and 208 times at 1.0 M NaCl, respectively, compared with the corresponding values of the control strain XCG002. As a result, the membrane integrity, cell growth, and cell survival rate of strain XCG010 increased by 24.4% ± 1.0%, 21.9% ± 1.5%, and 22.1% ± 1.1% at 1.0 M NaCl, respectively, compared with the corresponding values of the control strain XCG002 (wild-type strain with a control plasmid). These findings provided a novel strategy for engineering complex sphingolipids to enhance osmotic tolerance.

**IMPORTANCE** This study demonstrated a novel strategy for the manipulation of membrane complex sphingolipids to enhance S. cerevisiae tolerance to osmotic stress. Elo2, a sphingolipid acyl chain elongase, was related to osmotic tolerance through transcriptome analysis of the wild-type strain and an osmosis-tolerant strain generated from ALE. Overexpression of *ELO2* increased the content of complex sphingolipid with longer acyl chain; thus, membrane integrity and osmotic tolerance improved.

## Abstract (precis)

This study demonstrated a novel strategy for the manipulation of membrane complex sphingolipids to enhance S. cerevisiae tolerance to osmotic stress. Elo2, a sphingolipid acyl chain elongase, was related to osmotic tolerance through transcriptome analysis of the wild-type strain and an osmosis-tolerant strain generated from ALE. Overexpression of *ELO2* increased the content of complex sphingolipid with longer acyl chain; thus, membrane integrity and osmotic tolerance improved.

## INTRODUCTION

The growth performance (cell density and growth rate) of industrial strains is a key factor affecting the efficiency of the fermentation process (1). During industrial fermentation, the cell density and growth rate declines when industrial strains are subjected to harsh environmental conditions, including osmotic, pH, and oxidation stresses, which can cause an adverse biological and physiological response to the industrial strains (2, 3). The cell membrane is a natural barrier separating the extracellular environment from the intracellular components (4). Therefore, improving membrane function is a potential strategy to enhance the growth performance of industrial strains under harsh industrial conditions (5–7).

The manipulation of membrane lipids, such as phospholipids, sphingolipids, and sterols, is a primary and efficient strategy to enhance membrane function (8). Based on the structure of the phospholipids, one engineering strategy is to modulate the phospholipid head groups by altering the expression of the key phospholipid biosynthesis enzymes (9). For instance, when PssA, a phosphatidylserine synthase, was overexpressed, phosphatidylethanolamine (PE) content increased and membrane integrity enhanced; as a result, biorenewable fuel tolerance and titer were improved (9). Another strategy is to regulate the phospholipid fatty acid tails by changing the fatty acid length, increasing the ratio of saturated to unsaturated fatty acids, and producing transunsaturated fatty acids (tufa) (10–12). For example, through the expression of *cis-trans* isomerase (Cti) from Pseudomonas aeruginosa, tufa was incorporated into the Escherichia coli membrane, decreasing membrane fluidity; as a result, robustness and the biorenewable fuel titer were improved (13). The content and composition of sterols can be changed by engineering the key enzymes associated with sterol biosynthesis or by changing the transcription level of the sterol biosynthesis enzymes, which are affected by global transcription factors, such as Upc2 and Ecm22 (14). For example, the expression of a key sterol C-5 desaturase, FvC5SD, from an edible mushroom, in fission yeast improved the contents of ergosterol and oleic acid, which resulted in enhanced tolerance to ethanol and high temperature (15). Sphingolipids, which are signaling molecules, modulate cellular functions and fate, including cell division, cell death, life span, and autophagy (11, 16). Sphingolipids in the plasma membrane can help cells tolerate stress by manipulating the target of rapamycin complex 1 (TORC1), the sphingosine backbone, and the acyl chain (17, 18). For example, when mouse sphingomyelin synthase 1 (Sms1) was expressed in yeast, endogenous sphingolipids accumulated, and as a result, the tolerance of the strain to oxidation, osmotic, and temperature stresses improved (17). Some attempts were made to change sphingolipid content by metabolic engineering or the simulation of molecular dynamics (18–20). An increase in sphingolipids with very long fatty acyl chains in Zygosaccharomyces bailii made the membrane thicker and denser, which increased the free energy barrier for the permeation of acetic acid through the membrane and improved acetic acid resistance (18). These findings highlight the importance of developing novel strategies to improve stress resistance by engineering complex sphingolipids.

In this study, a mutant, XCG001, was obtained through adaptive laboratory evolution (ALE), and transcriptome sequencing (RNA-seq) analysis suggested that the mRNA level of *ELO2*, which is involved in the biosynthesis of very long fatty acids, was differentially upregulated in mutant XCG001. *ELO2* then was overexpressed through metabolic engineering and changed contents of fatty acids, phospholipids, and complex sphingolipids, leading to the improvement of cell membrane integrity, a result of which was increased osmotic tolerance.

## RESULTS

### Global transcriptome analysis of the mutant XCG001 and the wild-type strain at 0 M and 1.5 M NaCl.

**FIG 1:**
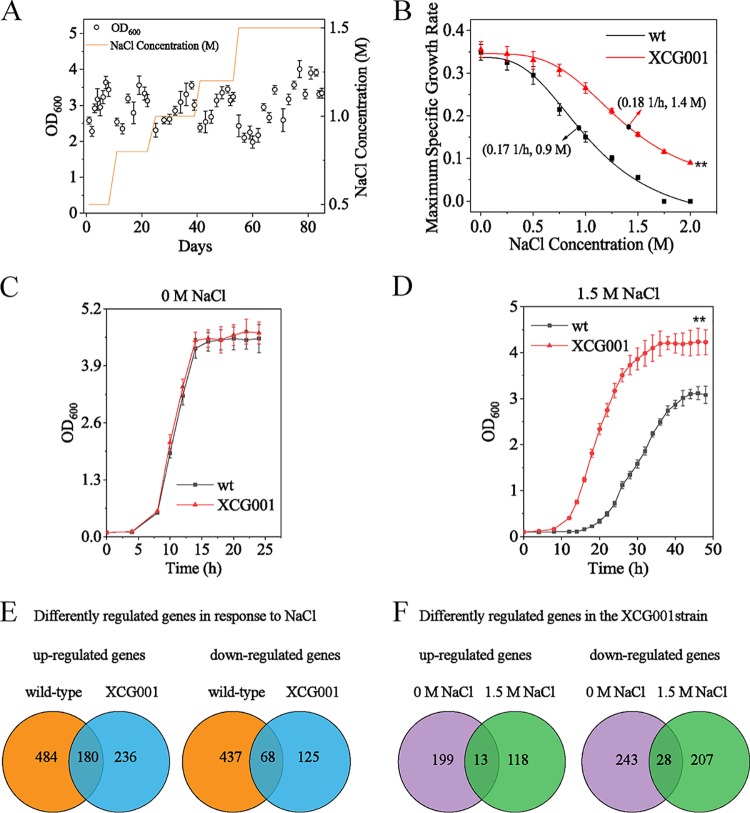
Global transcriptome analysis of the adaptive laboratory evolution (ALE) mutant XCG001 and the wild-type strain. (A) Cell growth trajectory showing changes in fitness during ALE in YNB medium with different concentrations of NaCl. The concentration of NaCl was stepwise improved from 0.5 to 1.5 M over time (orange line). (B) Maximum specific exponential growth rates of the wild-type strain and mutant XCG001 in YNB medium supplemented with increasing concentrations of salt. The half-maximal inhibitory concentration (IC_50_) was calculated by using the fitting curve of the data. (C) Growth profiles of mutant XCG001 and the wild-type strain in YNB medium under the 0 M NaCl condition. (D) Growth profiles of mutant XCG001 and the wild-type strain in YNB medium under the 1.5 M NaCl condition. (E) Venn diagrams depicting the numbers of upregulated and downregulated genes in the wild-type strain and mutant XCG001 under the 1.5 M NaCl condition compared with those genes’ expression levels in the corresponding strains under the 0 M NaCl condition. (F) Numbers of upregulated and downregulated genes in mutant XCG001 relative to their expression in the wild-type strain under 0 M and 1.5 M NaCl conditions.

To understand how S. cerevisiae adapts to higher osmotic stress, ALE was utilized to generate osmosis-tolerant mutants. As shown in Fig. 1A, the concentration of NaCl was increased with time in a stepwise fashion, reaching 1.5 M. After 300 generations of ALE, a clone (mutant XCG001) was isolated from the evolved population. The osmotic sensitivity of the wild-type strain and mutant XCG001 was tested. The half-maximal inhibitory concentration (IC_50_) values of the wild-type strain and mutant XCG001 were 0.9 M and 1.4 M NaCl, respectively (Fig. 1B). At 0 M NaCl, the final biomass of mutant XCG001 was similar to that of the wild-type strain, whereas at 1.5 M NaCl, the final biomass of mutant XCG001 increased by 37.3% ± 1.5% compared with that of the wild-type strain (Fig. 1C and D).

To identify the differentially regulated genes contributing to osmotic tolerance in mutant XCG001, transcriptome sequencing (RNA-seq) was conducted to compare global gene expression in mutant XCG001 and the wild-type strain at 0 M and 1.5 M NaCl. The restrictive thresholds of significantly expressed genes were used to screen the genes. First, the differentially expressed genes were analyzed at 1.5 M NaCl relative to 0 M NaCl in both the wild-type strain and mutant XCG001 (Fig. 1E). Transcriptional profiling analysis revealed that the expression levels of 1,169 genes were significantly changed in the wild-type strain, where 664 genes were upregulated and 505 genes were downregulated. In mutant XCG001, the expression levels of 609 genes displayed differential expression, where 416 genes were upregulated and 193 genes were downregulated. Additionally, 180 upregulated and 68 downregulated genes were common to both strains. Gene Ontology (GO) analysis indicated that the commonly upregulated genes were involved in glycolysis/gluconeogenesis, pyruvate metabolism, lipid metabolism, signaling transduction, fructose, and mannose metabolism. On the other hand, 68 downregulated genes were involved in the ribosome and amino acid metabolism (see Data Sets S1 and S2 in the supplemental material).

The significantly expressed genes in mutant XCG001, relative to those in the wild-type strain, then were analyzed at both 0 M and 1.5 M NaCl (Fig. 1F). At 0 M NaCl, the expression levels of 212 genes were upregulated and 271 genes were downregulated. At 1.5 M NaCl, 131 genes were upregulated and 235 genes were downregulated. These 131 upregulated genes include 13 genes that were commonly upregulated at 0 M and 1.5 M NaCl, and 118 genes were significantly upregulated only at 1.5 M NaCl. Based on the GO analysis, these 118 genes were involved in the steroid biosynthesis process, pentose-phosphate shunt, translation, regulation of transcription, phosphate ion transport, and response to stress. Moreover, 13 commonly upregulated genes were involved in transport, pyrimidine metabolism, and lipid metabolism, whereas 28 commonly downregulated genes were involved in pyruvate metabolism and transport (Data Sets S3 and S4). These results suggested that mutant XCG001 strengthened transport, pyrimidine metabolism, and lipid metabolism, which contribute to osmotic tolerance.

### Overexpression of *ELO2* enhanced osmotic tolerance.

**FIG 2:**
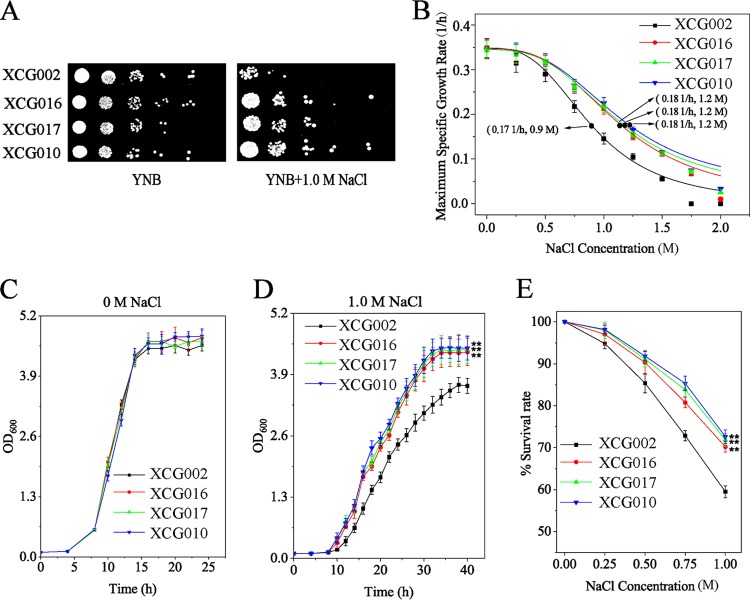
Overexpression of *ELO2* enhanced osmotic tolerance. (A) Control strains XCG002 (wild-type strain with a control plasmid pY13), P*_ADH1_-ELO2* (XCG016), P*_TDH3_-ELO2* (XCG017), and P*_TEF1_-ELO2* (XCG010) were spotted on YNB plates at 0 M and 1.0 M NaCl. (B) Maximum specific exponential growth rates of strains XCG002, XCG016, XCG017, and XCG010 in YNB supplemented with increasing NaCl concentrations. The half-maximal inhibitory concentration (IC_50_) was calculated by fitting the curve to the data. (C and D) Growth curves of strains XCG002, XCG016, XCG017, and XCG010 at 0 M and 1.0 M NaCl. (E) The survival rates of strains XCG002, XCG016, XCG017, and XCG010 over a range of NaCl doses (0.00, 0.25, 0.50, 0.75, and 1.00 M). All data are presented as mean values from three independent experiments. Error bars indicate the standard deviations. **, *P* < 0.01.

The mRNA levels of the 13 commonly upregulated genes were tested at 0 M, 1.0 M, and 1.5 M NaCl using quantitative reverse transcription-PCR (qRT-PCR) analysis (Table S1). At 0 M NaCl, mRNA levels of *FET4*, *ADH6*, *PHO89*, *EGT2*, *SAH1*, *ELO2*, *HXT4*, *SKG6*, *URA1*, *HXK2*, *YBL111C*, *RNR1*, and *SRL1* increased by 4.8-, 2.7-, 2.6-, 2.2-, 1.9-, 1.8-, 1.8-, 1.9-, 1.7-, 1.6-, 6.0-, 1.5-, and 1.5-fold, respectively, compared with the corresponding values of the wild-type strain. At 1.0 M NaCl, mRNA levels of *FET4*, *ADH6*, *PHO89*, *EGT2*, *SAH1*, *ELO2*, *HXT4*, *SKG6*, *URA1*, *HXK2*, and *SRL1* increased by 2.6-, 1.6-, 3.5-, 1.1-, 1.9-, 2.1-, 1.7-, 1.8-, 1.3-, 2.3-, and 1.6-fold, respectively, whereas *YBL111C* and *RNR1* decreased by 1.2- and 1.0-fold, respectively. Downregulated *YBL111C* and *RNR1* may decrease DNA replication for a high level of transcription and translation as a response to osmotic stress (21). At 1.5 M NaCl, mRNA levels of *FET4*, *ADH6*, *PHO89*, *EGT2*, *SAH1*, *ELO2*, *HXT4*, *SKG6*, *URA1*, *HXK2*, *YBL111C*, *RNR1*, and *SRL1* increased by 3.6-, 1.5-, 3.0-, 2.3-, 1.8-, 1.8-, 2.6-, 1.5-, 2.9-, 1.6-, 1.6-, 3.1-, and 2.5-fold, respectively. Furthermore, these genes were overexpressed in each strain, and the consequence on resistance to osmotic stress was evaluated (Fig. S2). Interestingly, only the overexpression of *ELO2* conferred resistance to osmotic stress. To confirm whether the expression of *ELO2* was positively correlated with osmotic tolerance, *ELO2* was overexpressed with two other constitutive promoters, P*_TDH3_* and P*_ADH1_* (promoter activity of P*_TDH3_* is weaker than that of P*_TEF1_* but stronger than that of P*_ADH1_* [22]). The spot results showed no obvious differences among the P*_ADH1_-ELO2* (XCG016), P*_TDH3_-ELO2* (XCG017), and P*_TEF1_-ELO2* (XCG010) strains at 1.0 M NaCl (Fig. 2A), and the IC_50_ values of strains XCG016, XCG017, and XCG010 were also equal (Fig. 2B). The growth curves of these four strains were different (Fig. 2C and D): at 0 M NaCl, the final biomasses of strains XCG016, XCG017, and XCG010 were similar to that of the control strain XCG002 (wild-type strain with control plasmid pY13), whereas at 1.0 M NaCl, the final biomass of strains XCG016, XCG017, and XCG010 improved by 19.1% ± 0.7%, 20.8% ± 1.3%, and 21.9% ± 1.5%, respectively, compared with the corresponding value of the control strain XCG002 (Fig. 2C and D). In addition, the survival rates were generated for the four strains over a broad concentration range of NaCl (Fig. 2E). At 1.0 M NaCl, the survival rate of the control strain XCG002 was 59.5% ± 1.1%, while the survival rates of strains XCG016, XCG017, and XCG010 were 70.2% ± 1.2%, 71.9% ± 1.5%, and 72.6% ± 1.8%, indicating approximate increases of 18.0% ± 0.9%, 20.8% ± 0.6%, and 22.1% ± 1.1%, respectively. These results suggested that the overexpression of *ELO2* enhanced the osmotic tolerance of S. cerevisiae.

### Overexpression of *ELO2* enhanced very long fatty acid contents.

**FIG 3:**
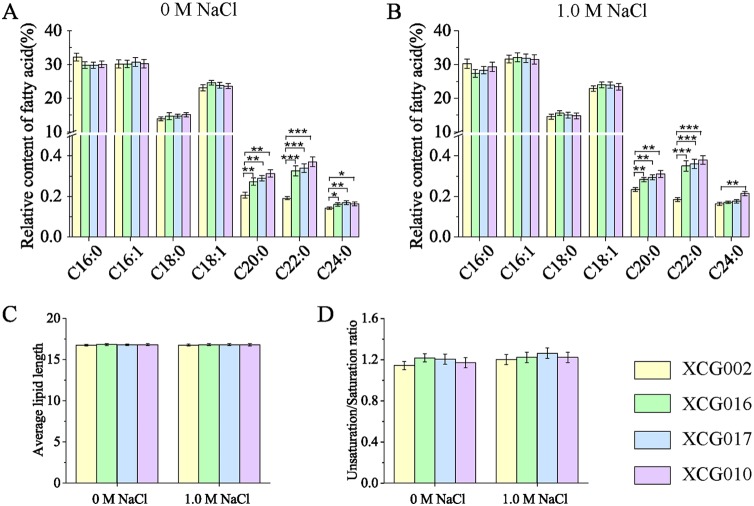
Overexpression of *ELO2* enhanced very long fatty acid content. (A) Fatty acid contents in strains XCG002, XCG016, XCG017, and XCG010 at 0 M NaCl. (B) Fatty acid contents in strains XCG002, XCG016, XCG017, and XCG010 at 1.0 M NaCl. (C) The fatty acid average length of strains XCG002, XCG016, XCG017, and XCG010 at 0 M and 1.0 M NaCl. (D) Unsaturation/saturation ratio of strains XCG002, XCG016, XCG017, and XCG010 at 0 M and 1.0 M NaCl. All data are presented as mean values from three independent experiments. Error bars indicate the standard deviations. *, *P* < 0.05; **, *P* < 0.01; ***, *P* < 0.001.

The fatty acid contents of the strains XCG016, XCG017, XCG010, and XCG002 were analyzed using gas chromatography. It was found that the contents of membrane fatty acids in strains XCG016, XCG017, and XCG010 were altered, especially that of C_22:0_ (Fig. 3A and B). At 0 M NaCl, the contents of C_20:0_, C_22:0_, and C_24:0_ in strain XCG010 increased by 52.3%, 94.1%, and 14.4%, respectively, compared with the corresponding values of the control strain XCG002, whereas the contents of C_16:0_, C_16:1_, C_18:0_, and C_18:1_ remained the same. At 1.0 M NaCl, the contents of C_20:0_, C_22:0_, and C_24:0_ in strain XCG010 increased by 33.1%, 106.4%, and 31.5%, respectively, while the contents of C_16:0_, C_16:1_, C_18:0_, and C_18:1_ remained unchanged. All of the fatty acid contents in strains XCG016 and XCG017 were similar to those in strain XCG010 at 0 M or 1.0 M NaCl (Fig. 3A and B).

The average fatty acid length in strains XCG016, XCG017, and XCG010 was equal to that of the control strain XCG002 at 0 M or 1.0 M NaCl, suggesting that the membrane “thickness” was not affected (Fig. 3C). The reason for unchanged membrane thickness may be that the proportion of C_20:0_ and C_22:0_ contents to the total fatty acid contents was only approximately 0.5%. In addition, the fatty acid unsaturation/saturation ratio did not increase in strain XCG016, XCG017, or XCG010 at 0 M or 1.0 M NaCl (Fig. 3D).

### Overexpression of *ELO2* altered complex sphingolipid contents.

**FIG 4:**
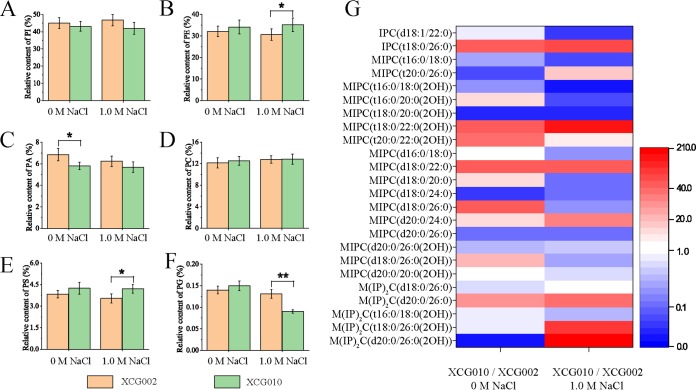
Overexpression of *ELO2* changed complex sphingolipid contents. (A to F) Phospholipid content (including phosphatidylserine [PS], phosphatidic acid [PA], phosphatidylinositol [PI], phosphocholine [PC], phosphatidylglycerol [PG], and phosphatidylethanolamine [PE]) changed in strain XCG002 and strain XCG010 at 0 M and 1.0 M NaCl. (G) The ratio of complex sphingolipid (including phosphorylceramide [IPC], mannosylinositol phosphorylceramide [MIPC], mannosyldiinositol phosphorylceramide [M(IP)_2_C] content in strain XCG010) to that of control strain XCG002 changed at 0 M and 1.0 M NaCl. All data are presented as mean values from three independent experiments. Error bars indicate the standard deviations. *, *P* < 0.05; **, *P* < 0.01.

The effect of overexpression of *ELO2* on the contents of phospholipids and complex sphingolipids in strain XCG010 was analyzed (Fig. 4A to G). It was found that overexpression of *ELO2* can change contents of the phospholipid (Fig. 4A to F) and complex sphingolipid (Fig. 4G). At 0 M NaCl, the content of phosphatidic acid (PA) decreased by 15.2%, but the contents of phosphatidylinositol (PI), phosphatidylethanolamine (PE), phosphocholine (PC), phosphatidylserine (PS), and phosphatidylglycerol (PG) in strain XCG010 remained unchanged. At 1.0 M NaCl, the contents of PE and PS in strain XCG010 increased by 18.9% and 15.0%, respectively, but the contents of PI, PA, and PC remained unchanged, and the content of PG decreased by 40.0%, compared with that of the control strain XCG002. At 0 M NaCl, the contents of IPC (t18:0/26:0), MIPC [t18:0/22:0(2OH)], MIPC [t20:0/22:0(2OH)], MIPC [d18:0/22:0], and MIPC [d18:0/26:0] in strain XCG010 increased 4,868.9%, 4,552.7%, 3,111.2%, 5,500.4%, and 4,079.1%, respectively, whereas the contents of MIPC (t20:0/26:0), MIPC [t18:0/20:0(2OH)], MIPC (d18:0/24:0), and M(IP)_2_C [d20:0/26:0(2OH)] in strain XCG010 decreased by 96.5%, 99.1%, 97.3%, and 99.7%, respectively, compared with the corresponding values of the control strain XCG002. At 1.0 M NaCl, the contents of IPC (t18:0/26:0), MIPC [t18:0/22:0(2OH)], MIPC (d18:0/22:0), MIPC (d20:0/24:0), M(IP)_2_C (d20:0/26:0), M(IP)_2_C [t18:0/26:0(2OH)], and M(IP)_2_C [d20:0/26:0(2OH)] in strain XCG010 increased by 8,833.4%, 16,689.4%, 6,329.2%, 2,391.1%, 2,792.8%, 11,376.4%, and 20,806.3%, respectively, whereas the contents of IPC (d18:1/22:0), MIPC (t16:0/18:0), MIPC [t16:0/18:0(2OH)], MIPC [t16:0/20:0(2OH)], MIPC [t18:0/20:0(2OH)], and MIPC (d20:0/26:0) in strain XCG010 decreased by 96.7%, 96.3%, 99.7%, 95.7%, 99.3%, and 88.0%, respectively, compared with the corresponding values of the control strain XCG002. These results suggested that a high level of complex sphingolipids with longer acyl chains enhances osmotic tolerance.

### Complex sphingolipids improve osmotic tolerance.

**FIG 5:**
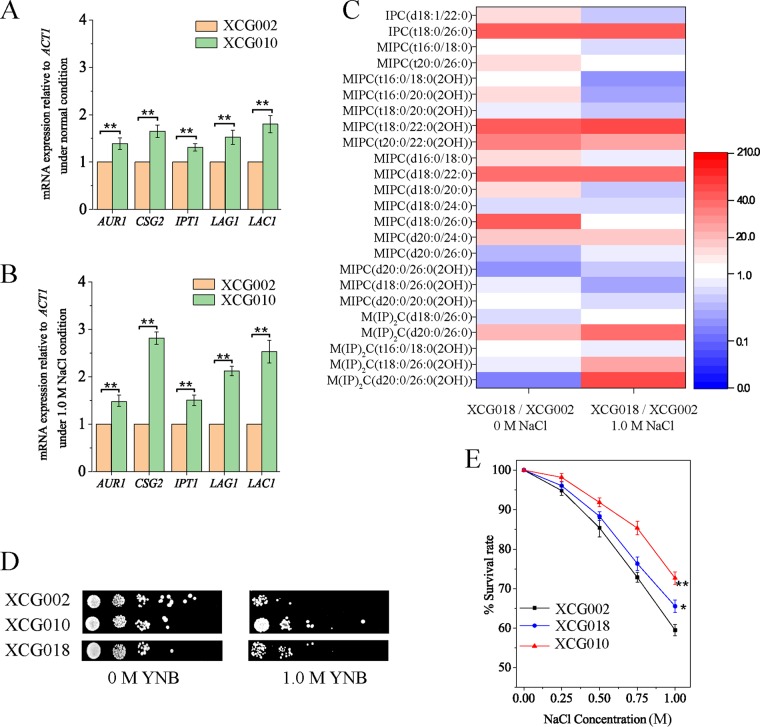
Complex sphingolipid improved osmotic tolerance. (A) The mRNA level of the complex sphingolipid biosynthesis genes in strains XCG002 and XCG010 at 0 M NaCl. (B) The mRNA level of complex sphingolipid biosynthesis genes in strains XCG002 and XCG010 at 1.0 M NaCl. (C) The ratio of complex sphingolipid content in strain XCG018 (deletion of *LAC1* in strain XCG010) to that of the control strain XCG002 changed at 0 M and 1.0 M NaCl. (D) Strains XCG002, XCG010, and XCG018 were spotted on plates containing or not containing 1.0 M NaCl. (E) The survival rates of strains XCG002, XCG010, and XCG018 over a range of NaCl doses (0.00, 0.25, 0.50, 0.75, and 1.00 M). All data are presented as mean values from three independent experiments. Error bars indicate the standard deviations. *, *P* < 0.05; **, *P* < 0.01.

To validate whether the increase in complex sphingolipid contents enhanced osmotic tolerance, the genetic details of strain XCG010 were investigated. The mRNA expression level of the complex sphingolipid biosynthesis genes in strains XCG010 and XCG002 was compared at 0 M and 1.0 M NaCl. At 0 M NaCl, the mRNA levels of *AUR1*, *CSG2*, *IPT1*, *LAG1*, and *LAC1* in strain XCG010 increased by 1.4- ± 0.12-, 1.7- ± 0.13-, 1.3- ± 0.08-, 1.5- ± 0.15-, and 1.8- ± 0.17-fold, respectively, compared to the corresponding values of the control strain XCG002 (Fig. 5A). At 1.0 M NaCl, the mRNA levels of *AUR1*, *CSG2*, *IPT1*, *LAG1*, and *LAC1* in strain XCG010 increased by 1.5- ± 0.12-, 2.8- ± 0.13-, 1.5- ± 0.11-, 2.1- ± 0.10-, and 2.5- ± 0.24-fold, respectively, compared to the corresponding values of the control strain XCG002 (Fig. 5B). These results are consistent with the high content of complex sphingolipids in strain XCG010. However, the mRNA level of the complex sphingolipid biosynthesis genes in strain XCG010 was different from the corresponding values in mutant XCG001 (Table S2). The reason for this may be that the comparison objects (strain XCG010 to XCG002 and mutant XCG001 to the wild-type strain) and conditions (under 1.0 M NaCl and 1.5 M NaCl) were different.

To evaluate whether the inhibition of complex sphingolipid biosynthesis affects the growth of strain XCG010, *LAC1*, which is involved in the synthesis of ceramide, was deleted to generate strain XCG018. The content of the complex sphingolipid in strain XCG018 was tested. At 0 M NaCl, the contents of IPC (t18:0/26:0), MIPC [t18:0/22:0(2OH)], MIPC [t20:0/22:0(2OH)], MIPC (d18:0/22:0), and MIPC (d18:0/26:0) in strain XCG018 increased by 3,921.3%, 4,029.7%, 2,392.7%, 3,512.4%, and 4,411.3%, respectively, compared with the corresponding values of the control strain XCG002. At 1.0 M NaCl, the contents of IPC (t18:0/26:0), MIPC [t18:0/22:0(2OH)], MIPC (d18:0/22:0), M(IP)_2_C (d20:0/26:0), M(IP)_2_C [t18:0/26:0(2OH)], and M(IP)_2_C [d20:0/26:0(2OH)] in strain XCG018 increased by 4,758.7%, 8,854.8%, 3,811.2%, 2,598.4%, 1,284.3%, and 8,745.1%, respectively, compared with the corresponding values of the control strain XCG002 (Fig. 5C). The spot results indicated that at 1.0 M NaCl, the growth of strain XCG018 was better than that of control strain XCG002 but worse than that of strain XCG010 (Fig. 5D). Moreover, at 1.0 M NaCl the survival rate of strain XCG018 (65.6% ± 2.1%) increased by 10.2% ± 0.2% compared with that of the control strain XCG002 (59.5% ± 1.1%) (Fig. 5E). These results suggested that an increase in complex sphingolipids is crucial for S. cerevisiae osmotic tolerance.

### Increased content of complex sphingolipid improved membrane integrity.

**FIG 6:**
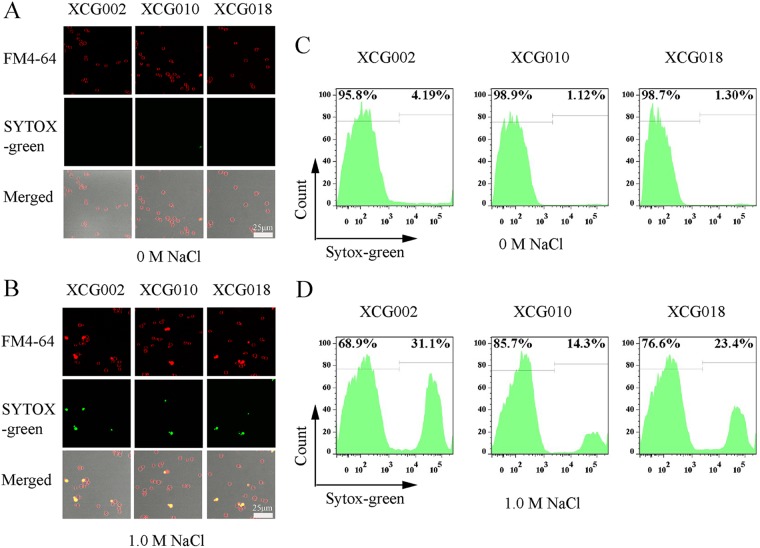
Increased complex sphingolipids changed membrane integrity. (A and B) Fluorescent microscopy analysis of membrane integrity in the XCG002, XCG010, and XCG018 cells at 0 M (A) or 1.0 M NaCl (B). Under the view of a confocal fluorescence microscope, all cells showed red fluorescence with an integral membrane, while only cells with a damaged membrane showed green fluorescence. Cells with a damaged membrane can be stained by SYTOX green, and cells with integral or damaged membrane all can be stained by FM4-64. The scale is 25 μm. (C and D) Flow cytometry analysis of membrane integrity in strains XCG002, XCG010, and XCG018 at 0 M or 1.0 M NaCl. All data are presented as mean values from three independent experiments.

To investigate the effect of complex sphingolipids on membrane integrity, strains XCG002, XCG010, and XCG018 were treated with 0 M or 1.0 M NaCl for 4 h and subjected to SYTOX green and FM4-64 uptake analysis. As illustrated in Fig. 6, at 0 M NaCl, the fluorescence microscope showed that almost all the cells of strains XCG002, XCG010, and XCG018 exhibited an integral membrane (Fig. 6A), whereas at 1.0 M NaCl, the number of cells with an integral membrane for strain XCG018 was more than that for the control strain XCG002 but less than that for strain XCG010 (Fig. 6B). The cells of strains XCG002, XCG010, and XCG018 were further analyzed using flow cytometry. At 0 M NaCl, the percentage of cells with an integral membrane of strains XCG010 and XCG018 was similar to that of the control strain XCG002, whereas at 1.0 M NaCl, the percentage of cells with an integral membrane for strain XCG010 (85.7% ± 3.2%) and XCG018 (76.6% ± 2.9%) increased by 24.4% ± 1.0% and 11.2% ± 0.4%, respectively, compared with that of the control strain XCG002 (68.9% ± 2.9%). These results suggested that an increase in complex sphingolipid content improved membrane integrity.

## DISCUSSION

S. cerevisiae is a well-established microorganism that is widely used for the industrial production of fine chemicals, such as organic acids and amino acids, which cause the low pH of the fermentation broth (1). To modulate a suitable pH for a medium, some alkaline reagents need to be added, which leads to osmotic stress (23). To elucidate the physiological mechanism of the cell membrane in osmotic resistance, RNA-seq analysis of the osmosis-tolerant mutant XCG001, obtained through ALE, found *ELO2* was associated with osmotic tolerance. Overexpression of *ELO2* enhanced the content of complex sphingolipids. The increased lipid content, as mentioned above, contributed to an improvement of membrane integrity, and, as a result, osmotic resistance increased.

ALE is a very efficient way to improve the phenotype of an industrial strain (24). For example, ALE has been used to increase the specific growth rate for the deletion of some genes in S. cerevisiae or genome-reduced E. coli with glucose as the energy source (25, 26) or enhance *Schizochytrium* species tolerance to high-salinity stress (27). After securing the ALE strains, an important objective was to further identify the targets for genetic modification. Three omics tools were applied for this purpose: genomics, transcriptomics, and metabolomics (25, 27, 28). For example, the growth of S. cerevisiae on glycerol was increased via ALE, and the transcriptome data revealed that the genes that were related to the tricarboxylic acid cycle and oxidative phosphorylation contributed to the increased growth (28). In this study, the evolutionary effect on the expression of the osmotic stress-related genes was divided into two aspects: (i) upregulated pathways, i.e., glycerol metabolism and ion transport were upregulated in mutant XCG001 under 1.5 M NaCl, as the expression levels of genes encoding glycerol-3-phosphate dehydrogenase (*GPD1*) and Na^+^-exporting P-type ATPase (*ENA1*) were upregulated 1.3- and 0.9-fold in mutant XCG001, respectively, compared with the corresponding values of the wild-type strain at 1.5 M NaCl; and (ii) without influencing the pathway, i.e., trehalose metabolism had no influence in mutant XCG001 under 1.5 M NaCl, as the expression level of the gene encoding trehalose-phosphatase (*TPS2*) was upregulated 0.1-fold (see Table S3 in the supplemental material). Glycerol as a major osmolyte can improve intracellular osmotic pressure, and, as a result, osmotic tolerance is improved (29). Na^+^-exporting P-type ATPase is one of the most important ion transports that can pump Na^+^ out of the cell to maintain osmotic pressure (30). However, trehalose synthesis was also unchanged in a yeast-like fungus under osmotic stress (31). Therefore, upregulated glycerol metabolism and ion transport may account for part of the increased osmotic resistance of mutant XCG001. Moreover, RNA-seq analysis of mutant XCG001 and the wild-type strain suggested that *ELO2* of 13 commonly upregulated genes was associated with osmotic tolerance. Elo2 is a fatty acid elongase that catalyzes C_16_-carbon fatty acids to C_22_, and its mutations have regulatory effects on 1,3-beta-glucan synthase, vacuolar ATPase, and the secretory pathway (32). *ELO2* overexpression may be caused by the changes of the sequence of *ELO2* promoter and transcription factors (TFs) due to evolution under osmotic conditions. *ELO2* overexpression could be controlled by downregulated negative TFs or upregulated positive TFs. For example, when the expression of *YY1* (a negative TF of *ELO2*) decreased due to the change of *YY1* sequence, less *YY1* can bind to the promoter of *ELO2*, which could lead to *ELO2* overexpression (33).

The overexpression of *ELO2* changed the lipid composition, including that of fatty acids, phospholipids, and complex sphingolipids. Sphingolipids play an important role in physiological functions by regulating cell growth and responding to environmental stress (34). The effect of sphingolipid synthesis on environmental stress can be divided into two aspects: (i) the overexpression or knockout of the sphingolipid synthesis gene (17, 35, 36), for example, when *SUR1* was deleted in S. cerevisiae, mutant *sur1* was sensitive to Ca^2+^ (35), and (ii) the addition of a certain sphingolipid, for instance, the addition of phytosphingosine or glucosylceramide or sphingolipid long-chain bases enhanced tolerance to environmental stress (34, 37, 38). Furthermore, lipid composition and content may undergo changes because of the metabolic pathway genes, harsh environmental conditions, and transcription factors (11, 39). The manipulation of lipid biosynthesis genes can change lipid content. *ELO3*, an *ELO2* paralog, is related to biosynthesis of sphingolipid with a C_26_ acyl chain (32). Environmental or chemical stresses affect lipid metabolism, which plays a role in maintaining membrane homeostasis and cell growth. A case in point is the membrane unsaturated fatty acids to saturated fatty acids ratio being increased under high-pressure homogenization stress, which enables the strain to avoid damage (39). Transcription factors, such as Mga2, that enable changes in the expression of lipid biosynthesis genes may change the lipid composition indirectly (40).

In this study, the enhancement of the complex sphingolipid content increased the membrane integrity and osmotic tolerance of S. cerevisiae. Membrane integrity could be enhanced by engineering membrane components, including (i) transporter proteins, for example, when the sugar and ion transporter OmpF was deleted and the long-chain fatty acid transporter, FadL, was overexpressed in E. coli, the membrane integrity was enhanced and the fatty acid titer improved (41); (ii) phospholipids, i.e., membrane integrity can also be altered by modifying the distribution of phospholipid head groups, by adjusting phospholipid saturation, and by altering the phospholipid acyl chain length (11); (iii) sterols can modulate the membrane integrity to resist stress (42); and (iv) sphingolipids, i.e., when the sphingolipid biosynthesis genes were deleted in S. cerevisiae, the resultant strains exhibited resistance to amphipathic peptidomimetic, which decreased membrane integrity (43). The increased complex sphingolipids may change the raft structure to help osmotic tolerance in three ways: lipid-lipid interaction, lipid-protein interaction, and membrane fusion. Lipid-lipid interaction requires sterols and sphingolipids acting as functional pairs to help nanodomain formation on the membrane, and as a result, membrane stability increases (11). Lipid-protein interaction could help enhance the function and stability of GPI-anchored and transmembrane domains of proteins (44). Due to an intrinsic property of the very long acyl chain of sphingolipids, membrane fusion may get stimulated (45). Furthermore, Na^+^ may bind to sphingolipids to enhance osmotic tolerance through the calcium channel being activated directly or indirectly. The Ca^2+^ influx channel is directly activated by Na^+^ binding to sphingolipids, which activates Ca^2+^-binding proteins and upregulates the Na^+^/H^+^ antiporter to pump Na^+^ out of the cell (46). The calcium channel is activated indirectly through Na^+^ binding to sphingolipids to drive the formation of a microdomain on the membrane, which triggers the calcium signals and leads to osmosis-tolerant responses (47).

In summary, ALE was used to obtain an osmosis-tolerant strain, XCG001, and RNA-seq analysis of mutant XCG001 and the wild-type strain was used to identify a key gene, *ELO2*, associated with osmotic tolerance. Furthermore, overexpression of *ELO2* increased the content of complex sphingolipids with longer acyl chains. As a result, membrane integrity increased, and the osmotic resistance enhanced. This study provides a novel strategy to manipulate membrane complex sphingolipids to increase membrane integrity and osmotic tolerance.

## MATERIALS AND METHODS

### Strains and media.

All S. cerevisiae strains and plasmids used in this study are listed in Table 1. Plasmids pY131 and pY132 were constructed by replacing the promoter P*_TEF_* of pY13 with P*_ADH1_* and P*_TDH3_*, respectively. Overexpression strains were constructed using pY13, pY131, and pY132 plasmids carrying the target genes. All plasmids were transformed into yeast cells using the lithium acetate transformation method (48). Homologous recombination was used for *lac1* gene deletion. For the *LEU2* marker, the upstream and downstream regions of the target gene open reading frame were fused by fusion-PCR, and the PCR products were transformed into yeast cells using the lithium acetate transformation method. All primers used in this study are listed in Table 2. Yeast was cultivated in yeast extract peptone dextrose (YPD) medium and yeast nitrogen base (YNB) medium at 30°C with shaking at 200 rpm.

**TABLE 1:** Strains used in this study

Strain	Relevant characteristic	Source
Strains		
BY4741	*MAT***a** *hisΔ1 leu2Δ0 met15Δ0 uraΔ0*	This study
XCG001	ALE mutant of BY4741 that tolerates 1.5 M NaCl	This study
XCG002	BY4741 harboring pY13	This study
XCG003	BY4741 harboring pY13-FET4	This study
XCG004	BY4741 harboring pY13-ADH6	This study
XCG005	BY4741 harboring pY13-PHO89	This study
XCG006	BY4741 harboring pY13-EGT2	This study
XCG007	BY4741 harboring pY13-SAH1	This study
XCG008	BY4741 harboring pY13-HXT4	This study
XCG009	BY4741 harboring pY13-SKG6	This study
XCG010	BY4741 harboring pY13-ELO2	This study
XCG011	BY4741 harboring pY13-URA1	This study
XCG012	BY4741 harboring pY13-HXK2	This study
XCG013	BY4741 harboring pY13-YBL111C	This study
XCG014	BY4741 harboring pY13-RNR1	This study
XCG015	BY4741 harboring pY13-SRL1	This study
XCG017	BY4741 harboring pY131-ELO2	This study
XCG017	BY4741 harboring pY132-ELO2	This study
XCG018	Gene *lac1* deleted in strain XCG010	This study
Plasmids		
pY13	2μm, *Amp*, *HIS1*, P_TEF_	Laboratory storage
pY13-FET4	2μm, *Amp*, *HIS1*, P_TEF_-FET4	This study
pY13-ADH6	2μm, *Amp*, *HIS1*, P_TEF_-ADH6	This study
pY13-PHO89	2μm, *Amp*, *HIS1*, P_TEF_-PHO89	This study
pY13-EGT2	2μm, *Amp*, *HIS1*, P_TEF_-EGT2	This study
pY13-SAH1	2μm, *Amp*, *HIS1*, P_TEF_-SAH1	This study
pY13-HXT4	2μm, *Amp*, *HIS1*, P_TEF_-HXT4	This study
pY13-SKG6	2μm, *Amp*, *HIS1*, P_TEF_-SKG6	This study
pY13-ELO2	2μm, *Amp*, *HIS1*, P_TEF_-ELO2	This study
pY13-URA1	2μm, *Amp*, *HIS1*, P_TEF_-URA1	This study
pY13-HXK2	2μm, *Amp*, *HIS1*, P_TEF_-HXK2	This study
pY13-YBL111C	2μm, *Amp*, *HIS1*, P_TEF_-YBL111C	This study
pY13-RNR1	2μm, *Amp*, *HIS1*, P_TEF_-RNR1	This study
pY13-SRL1	2μm, *Amp*, *HIS1*, P_TEF_-SRL1	This study
pY131	Replace pY13 promoter P_TEF_ with promoter P_ADH1_	This study
pY132	Replace pY13 promoter P_TEF_ with promoter P_TDH3_	This study
pY131-ELO2	2μm, *Amp*, *HIS1*, P_ADH1_-ELO2	This study
pY132-ELO2	2μm, *Amp*, *HIS1*, P_TDH3_-ELO2	This study

**TABLE 2:** Primers used in this study

Primer function and name	Sequence (5′–3′)
Overexpression	
FET4-F1	TAGTGGATCCATGGGTAAAATTGCA
FET4-F2	ATGACTCGAGCTATTTTTCCAACATCATA
ADH6-F1	CTAGTGGATCCATGTCTTATCCTGAGAA
ADH6-F2	ATGACTCGAGCTAGTCTGAAAATTCT
PHO89-F1	TAGTGGATCCATGGCTTTACATCAA
PHO89-F2	ATGACTCGAGTTATGTCATTTGGTATTC
EGT2-F1	GCAGGAATTCATGAATAAACTATTGTTACATCT
EGT2-F2	ATGACTCGAGTTACAGCAGAAATGAGA
SAH1-F1	TAGTGGATCCATGTCTGCTCCAGCT
SAH1-F2	ATGACTCGAGTCAATATCTGTAGTGG
HXT4-F1	TAGTGGATCCATGTCTGAAGAAGCTG
HXT4-F2	ATGACTCGAGCTACTTTTTTCCGAAC
SKG6-F1	TAGTGGATCCATGTACCACACCCATA
SKG6-F2	ATGACTCGAGTCAGTTGACGGTATAATT
ELO2-F1	TAGTGGATCCATGAATTCACTCGTTAC
ELO2-F2	ATGACTCGAGTTACCTTTTTCTTCTGTG
URA1-F1	TAGTGGATCCATGACAGCCAGTTTAACTA
URA1-F2	ATGACTCGAGTTAAATGCTGTTCAACTT
HXK2-F1	TAGTGGATCCATGGTTCATTTAGGTCC
HXK2-F2	ATGACTCGAGTTAAGCACCGATGATAC
YBL111C-F1	TAGTGGATCCATGAAAGTTTCCGATAG
YBL111C-F2	ATGACTCGAGTCAGTGACAAACTCCT
RNR1-F1	TAGTGGATCCATGTACGTTTATAAAAGAGAC
RNR1-F2	ATGACTCGAGTTAACCCGAACACATTTC
SRL1-F1	TAGTGGATCCATGCTTCAATCCGTT
SRL1-F2	ATGACTCGAGTCACCAACTGGTCGAA
Replace promoter	
P_ADH1_-F1	GCTGGAGCTCATCCTTTTGTTGTTTCC
P_ADH1_-F2	GGATCCACTAGTTCTAGAAGTTGATTGTATGCTTGG
P_TDH3_-F1	GCTGGAGCTCTCATTATCAATACTGCCA
P_TDH3_-F2	CTAGTTCTAGATTTGTTTGTTTATGTGTGTTTATTC
Gene deletion	
L-lac1-F1	GAATGAAAAATAGTTGGAAAGGAAACA
L-lac1-F2	AGCTCTTGTTTATTGATACTGTGTC
Leu2 (lac1)-F1	AGTATCAATAAACAAGAGCTATGTCTGCCCCTAAGAAGAT
Leu2 (lac1)-F2	CTTAAAAACACCGTTTTCCTTTAAGCAAGGATTTTCTTAACTTCTTCG
R-lac1-F1	AGGAAAACGGTGTTTTTAAGTAGTA
R-lac1-F2	CATATTTAGTTTGCACTGAAGGAGAA
RT-PCR	
LAC1-F1	TTCACTTCTGGTAACACTA
LAC1-F2	CTAATAGCGAACGGTCTA
LAG1-F1	CTTGACTGGTGACTCTAA
LAG1-F2	TATGATATGGCTACGAACA
AUR1-F1	ATGGTCATACACTTCAAT
AUR1-F2	GGTTCATCAGTCATATTAAG
CSG2-F1	CAAGTGTAATAGGCTACG
CSG2-F2	AAGGTCAGATAGAAGGTTA
IPT1-F1	CATCTTCATTCACCGTAT
IPT1-F2	TTATTGCCATTGCTGTTA

### Adaptive laboratory evolution.

S. cerevisiae BY4741 was cultivated in 25 ml of YNB medium with histidine, leucine, methionine, uracil, and increasing salt concentrations in a 100-ml flask (0.5 M NaCl, 0.75 M NaCl, 1.0 M NaCl, 1.2 M NaCl, and 1.5 M NaCl). When the optical density at 600 nm (OD_600_) reached around 4, the strain was transferred to a new salt medium with an initial OD_600_ of approximately 0.1. The concentration of salt was increased when the maximum specific growth rate reached around 0.3.

### Spot assay.

Yeast cells were cultivated in the logarithmic phase and diluted to an OD_600_ of 1.0. Aliquots (4 μl) of 10-fold serial dilutions were spotted onto YNB agar plates with or without the indicated concentration of NaCl. Growth was assessed after incubation for 2 to 4 days at 30°C.

### IC_50_, growth curve, and survival rate.

Maximum exponential growth rates of yeast were determined in YNB supplemented with increasing salt concentrations. The half-maximal inhibitory concentration (IC_50_) was calculated by fitting a Hill-type model to the data. Data points and error bars represent means and standard deviations (SD) from three biological replicates. To test the growth curve of S. cerevisiae at different concentrations of NaCl, cells were cultivated in log phase and diluted into fresh YNB medium at an OD_600_ of 0.1 with different concentrations of NaCl. The OD_600_ values were recorded by taking determining curves at regular time intervals. Cell survival rates were assessed by log-phase cells treated with various concentrations of NaCl for 1 h at 30°C with shaking at 200 rpm. Cells next were diluted and plated on YNB agar plates with various concentrations of NaCl. After incubation for 2 to 4 days at 30°C, the surviving colonies were counted. The survival rates are expressed relative to that of untreated cells of the corresponding strain. The treatment level of NaCl was chosen according to the standard that the cell density of a strain growing to the stationary phase is similar to that of the wild-type strain at 0 NaCl. At 1.5 M NaCl, mutant XCG001 can grow well, and its cell density at stationary phase was close to that of the wild-type strain at 0 M NaCl (Fig. 1D). At 1.0 M NaCl, mutant XCG010 can grow well, and its cell density at stationary phase was close to that of the wild-type strain at 0 M NaCl (Fig. 2D). At 1.5 M NaCl, the cell density at the stationary phase in mutant XCG010 decreased by 12.7% compared with that of the wild-type strain at 0 M NaCl (see Fig. S3 in the supplemental material). Therefore, mutant XCG001 was treated with 1.5 M NaCl and mutant XCG010 was treated with 1.0 M NaCl.

### Transcriptome analysis.

The wild-type strain and mutant XCG001 were cultured in log phase at 0 M and 1.5 M NaCl. The collected strains were frozen at −80°C and sent to the Genewiz Institute for RNA extraction and global gene analysis.

### qRT-PCR analysis.

Total RNA was extracted using a MiniBEST universal RNA extraction kit, and 1 μg was taken to synthesize cDNA using the PrimeScript II first-strand cDNA synthesis kit (TaKaRa, Japan). The cDNA mixture was diluted to about 100 ng/μl and used as the template for gene expression level analysis by qRT-PCR. qRT-PCR was performed with TB green premix *Ex Taq* (TaKaRa Bio) using an iQ5 continuous fluorescence detector system (Bio-Rad, Hercules, CA). Data were normalized to that of the β-actin gene ACT1. The primer sequences for qRT-PCR are listed in Table 2.

### Fatty acid analysis.

Fatty acids of yeast were extracted using a NaOH-methanol-distilled water solution (3:10:10, wt/vol/vol) and freeze-dried. The dried sample then was treated with 2 ml boron trifluoride (BF_3_)-methanol (12:88, vol/vol) to produce fatty acid methyl esters, as described previously (49). Finally, samples were analyzed by gas chromatography (GC) with a polyethylene glycol capillary column, eluted at a flow rate of 29.6 ml/min and a column pressure of 63.4 kPa. Data analysis was based on the Supelco 37 standard (47885-U; Sigma). Fatty acid was determined according to GC analysis with fatty acid standards (Supelco 37) (Fig. S4).

### Phospholipid measure.

Phospholipids were extracted from the freeze-dried samples using chloroform-methanol as described previously (50). Dried phospholipids were obtained under a nitrogen stream and reconstituted in chloroform-methanol (1:1, vol/vol). Samples were analyzed by ultrahigh-performance liquid chromatography-tandem mass spectrometry (UPLC-MS; Waters, USA) with a CORTECS UPLC hydrophilic interaction liquid chromatography (HILIC) column (2.1 by 150 mm; inner diameter, 1.6 μm) with gradient elution at 45°C and a rate of infusion of 0.3 ml · min^−1^.

### Complex sphingolipid measure.

Strains were cultured in YNB medium with or without 1.0 M NaCl for 6 to 8 h and washed with phosphate-buffered saline (PBS). The cell pellets were lysed in PBS by bead-beating mechanical disruption at 4°C. The supernatants then were extracted with chloroform-methanol (2:1, vol/vol) at a final ratio of 20% (vol/vol). Centrifugation using a refrigerated centrifuge at 4°C was performed to obtain the supernatant. The extracts were evaporated to dryness under nitrogen at room temperature and stored at −80°C. The dried samples were sent to the Profleader Institute for complex sphingolipid analysis and solubilized in dichloromethane-methanol (2:1, vol/vol) before analysis by UHPLC quantitative time of flight mass spectrometry (Agilent) analysis (Fig. S5).

### Cell membrane integrity analysis.

Cell membrane integrity was analyzed by microscopy and flow cytometry. For microscopy analysis of cell membrane integrity, the log-phase cells were treated with 0 M and 1.0 M NaCl for 4 h and washed with PBS twice. The samples then were subjected to SYTOX green and FM4-64 uptake for 20 min, placed on a microscope slide, and covered with a coverslip (51–53). Images were acquired using a Nikon ECLIPSE 80i microscope equipped with a Nikon DS-Ri1 camera. For flow cytometry of cell membrane integrity, 10,000 counts of stained cells were recorded using a 0.5-ml s^−1^ flow rate. All data were exported in FCS3 format and processed using FlowJo software (FlowJo, LLC).

### Statistical analysis.

Experimental data are shown as the means ± standard errors of the means (SEM). All quantitative data were analyzed using Student's *t* test or one-way analysis of variance (ANOVA). Each experiment was repeated at least three times.

### Accession number(s).

The RNA-seq raw reads were submitted to NCBI under BioProject number PRJNA568205, and the Sequence Read Archive (SRA) entries are SRR10150286, SRR10150285, SRR10150284, and SRR10150283.

## Supplementary Material

Supplemental file 1

Supplemental file 2

Supplemental file 3

Supplemental file 4

Supplemental file 5
