# Supplementary material for: Enhancement of Sphingolipid Synthesis Improves Osmotic Tolerance of Saccharomyces cerevisiae
Source: Appl Environ Microbiol. 2020 Apr 1;86(8):e02911-19. doi: 10.1128/AEM.02911-19 (PMC7117927; doi:10.1128/AEM.02911-19)
Supplement: Supplemental file 1 [file AEM.02911-19-s0001.pdf]

**Supplementary Material**

# **Engineering membrane complex sphingolipid improve osmotic tolerance of *Saccharomyces cerevisiae***

Guoxing Zhu[a,b], Nannan Yin[a,b], Qiuling Luo[a,b], Jia Liu[a,b], Xiulai Chen[a,b], Liming Liu[a,b,#], Jianrong Wu[b,c,##]

[a] State Key Laboratory of Food Science and Technology, Jiangnan University, Wuxi 214122, China

[b] Key Laboratory of Industrial Biotechnology, Ministry of Education, Jiangnan University, Wuxi 214122, China

[c] Key Laboratory of Carbohydrate Chemistry and Biotechnology, Ministry of Education, School of Biotechnology, Jiangnan University, Wuxi 214122, China

[#] Address correspondence to: Liming Liu

[##] Address correspondence to: Jianrong Wu

E-mail: mingll@jiangnan.edu.cn (Liming Liu), kinowu@jiangnan.edu.cn (Jianrong Wu).

*Liming Liu and Jianrong Wu contributed equally to this work. Author order was determined by drawing straws.*

**1. Supplementary Data**

**Spreadsheet 1.** Up-regulated genes in the wild-type strain and mutant XCG001 at 1.5 M NaCl, respectively, compared with that of the wild-type strain and mutant XCG001 at 0 M NaCl.

**Spreadsheet 2.** Down-regulated genes in the wild-type strain and mutant XCG001 at 1.5 M NaCl, respectively, compared with that of the wild-type strain and mutant XCG001 at 0 M NaCl.

**Spreadsheet 3.** Up-regulated genes in mutant XCG001, compared with the wild-type strain at 0 M NaCl and at 1.5 M NaCl.

**Spreadsheet 4.** Down-regulated genes in mutant XCG001, compared with the wild-type strain at 0 M NaCl and at 1.5 M NaCl.

## 2. Supplementary Tables and Figures

### 2.1 Supplementary Tables

**Table S1 mRNA levels of commonly up-regulated 13 genes**. mRNA levels of commonly up-regulated 13 genes in mutant XCG001 relative to those of wild-type strain at 0 M, 1.0 M and 1.5 M NaCl.

| **Gene** | **mRNA levels (-fold)** | | | **Gene description** |
|---|---|---|---|---|
| | **0 M NaCl** | **1.0 M NaCl** | **1.5 M NaCl** | |
| *FET4* | 4.8 | 2.6 | 3.6 | Fe transporter |
| *ADH6* | 2.7 | 1.6 | 1.5 | Alcohol dehydrogenase |
| *PHO89* | 2.6 | 3.5 | 3.0 | Na+/Pi cotransporter |
| *EGT2* | 2.2 | 1.1 | 2.3 | Early G1 transcript |
| *SAH1* | 1.9 | 1.9 | 1.8 | Hydrolase |
| *ELO2* | 1.8 | 2.1 | 1.8 | Fatty acid elongase |
| *HXT4* | 1.8 | 1.7 | 2.6 | Hexose transporter |
| *SKG6* | 1.9 | 1.8 | 1.5 | Membrane protein |
| *URA1* | 1.7 | 1.3 | 2.9 | Dihydroorotate dehydrogenase |
| *HXK2* | 1.6 | 2.3 | 1.6 | Hexokinase |
| *YBL111C* | 6.0 | -1.2 | 1.6 | Hypothetical protein |
| *RNR1* | 1.5 | -1.0 | 3.1 | Reductase subunit |

| | | | | |
|---|---|---|---|---|
| *SRL1* | 1.5 | 1.6 | 2.5 | Mannoprotein |

**Table S2 mRNA levels of complex sphingolipid biosynthesis genes.** mRNA levels of complex sphingolipid biosynthesis genes were expressed in XCG001 relative to those of wild-type cells.

| **Genes** | **mRNA levels (-fold)** | |
|---|---|---|
| | **0 M NaCl** | **1.5 M NaCl** |
| *AUR1* | 1.2 | -1.1 |
| *CSG2* | 1.1 | 1.7 |
| *IPT1* | 1.8 | 1.7 |
| *LAG1* | -1.3 | 1.0 |
| *LAC1* | -1.8 | 1.1 |

**Table S3 Genes linked to the typical osmotic stress response were downregulated in the XCG001 strain compared with wild-type strain at 1.5 M NaCl.**

| Gene | mRNA levels (-fold) | Gene description |
|---|---|---|
| *ENA1* | 0.9 | Na(+)-exporting P-type ATPase |
| *ENA2* | 0.3 | Na(+)-exporting P-type ATPase |
| *ENA5* | 1.4 | Na(+)-exporting P-type ATPase |
| *GPD1* | 1.3 | glycerol-3-phosphate dehydrogenase |
| *GPD2* | 0.3 | glycerol-3-phosphate dehydrogenase |
| *TPS1* | -0.2 | alpha, alpha-trehalose-phosphate synthase |
| *TPS2* | 0.1 | trehalose-phosphatase |
| *TPS3* | 0.4 | trehalose 6-phosphate synthase |

**2.2 Supplementary Figures**

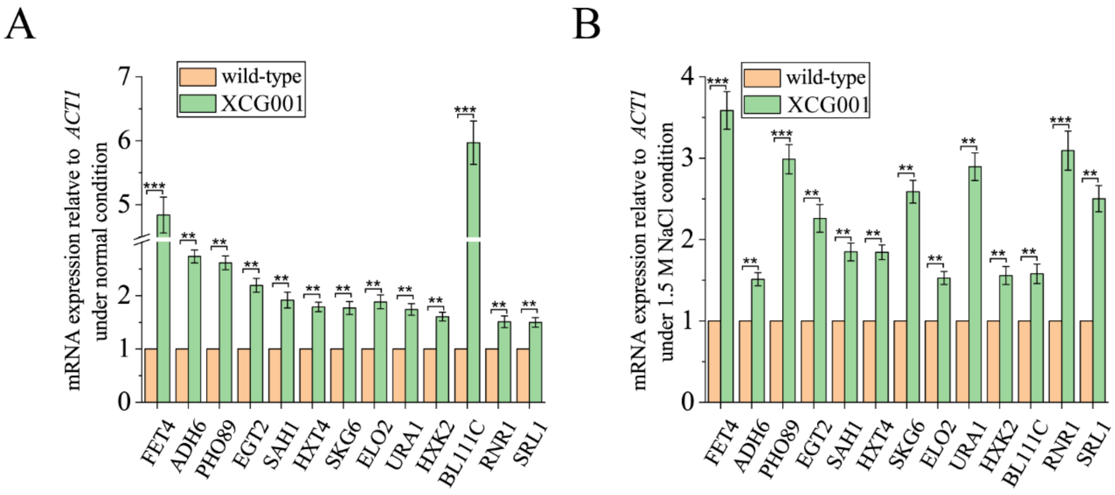


**Fig. S1. The mRNA levels of 13 genes in the wild-type strain and mutant XCG001.** (A) At 0 M NaCl, the mRNA levels of 13 genes in mutant XCG001 and the wild-type strain. (B) At 1.5 M NaCl, the mRNA levels of 13 genes in mutant XCG001 and the wild-type strain. All data are presented as mean values of three independent experiments. Error bars indicate the standard deviations. **, $P < 0.01$; ***, $P < 0.001$.

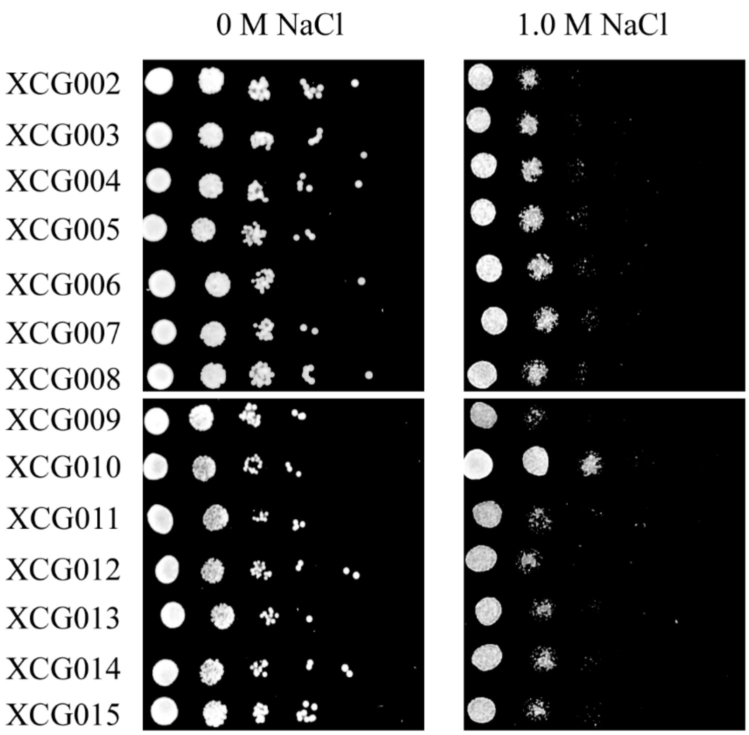


**Fig. S2. Growth profile of 13 mutant strains grown on YNB medium with or without 1.0 M NaCl.**

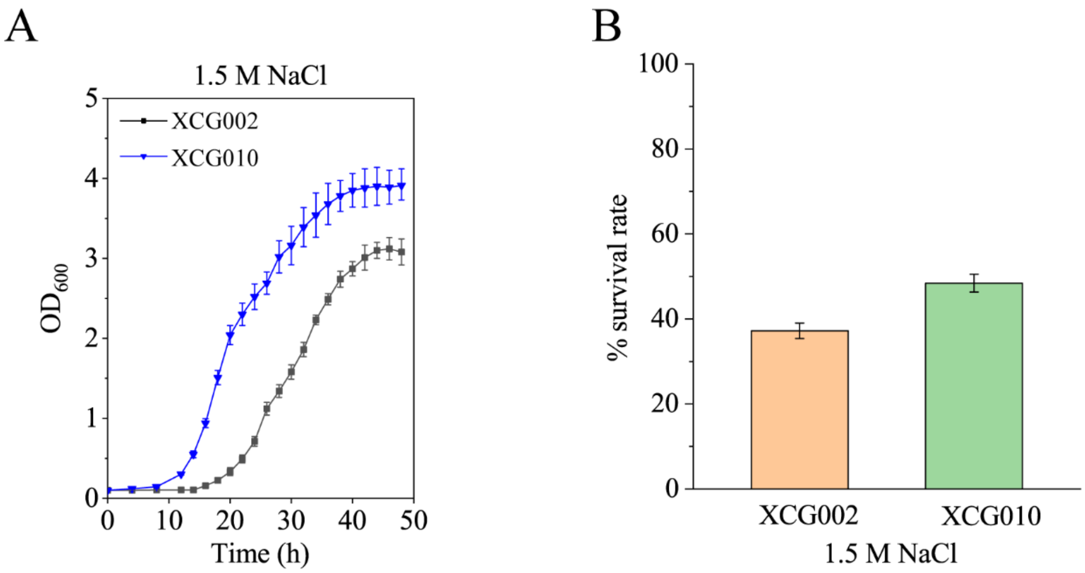


**Fig. S3 Growth profiles and survival rate of mutant XCG002 and XCG010 in YNB medium at 1.5 M NaCl.**

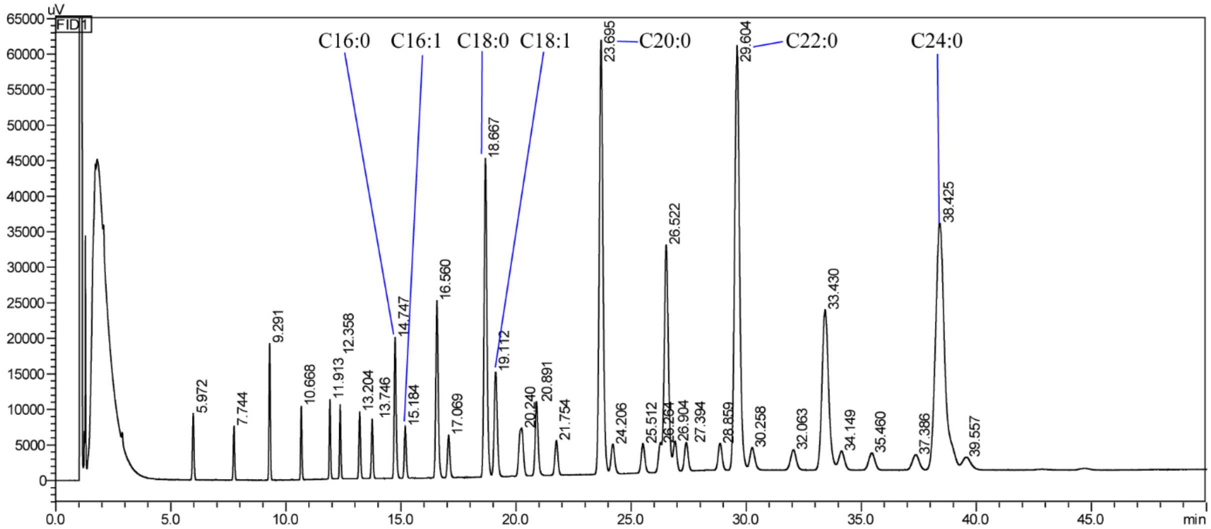


**Fig. S4. GC analysis of standard fatty acids.** Fatty acid was ensured by order of Supelco 37 retention time and weight percentage of each fatty acid accounts for Supelco 37.

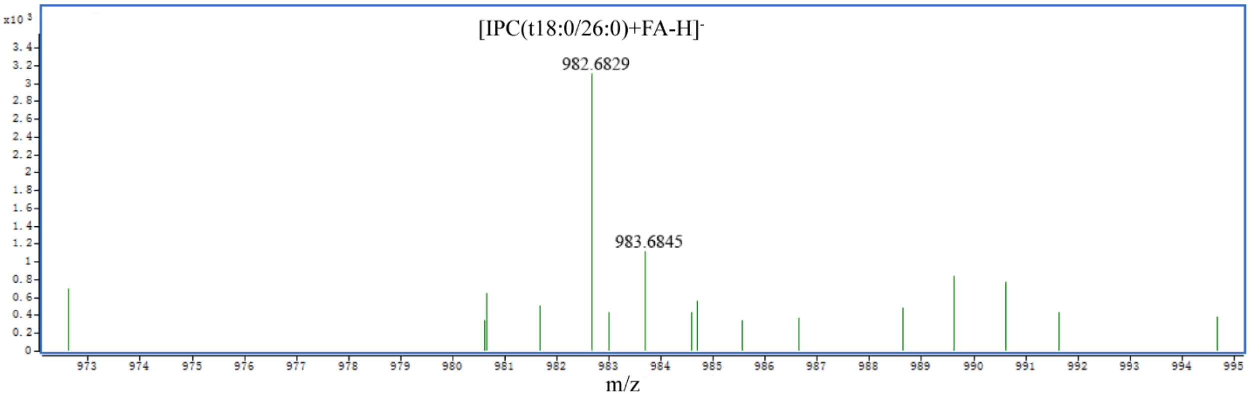
[IPC(t18:0/26:0)+FA-H]-
982.6829
983.6845
x10 3
m/z

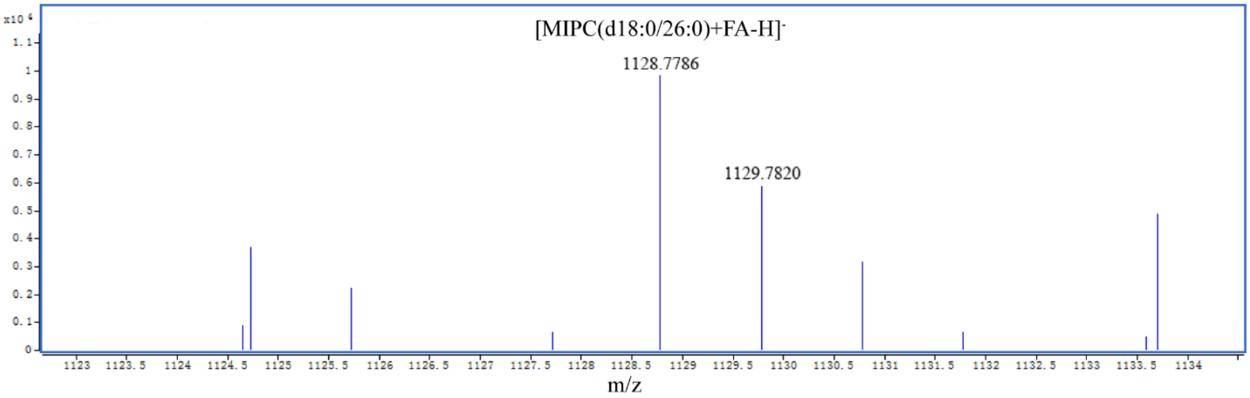
[MIPC(d18:0/26:0)+FA-H]-
1128.7786
1129.7820
x10 4
m/z

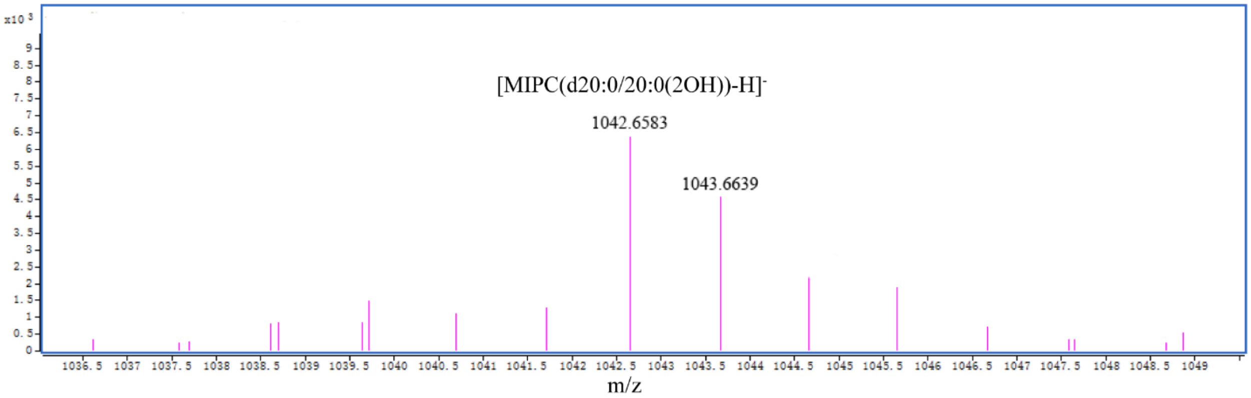
[MIPC(d20:0/20:0(2OH))-H]-
1042.6583
1043.6639
x10 3
m/z

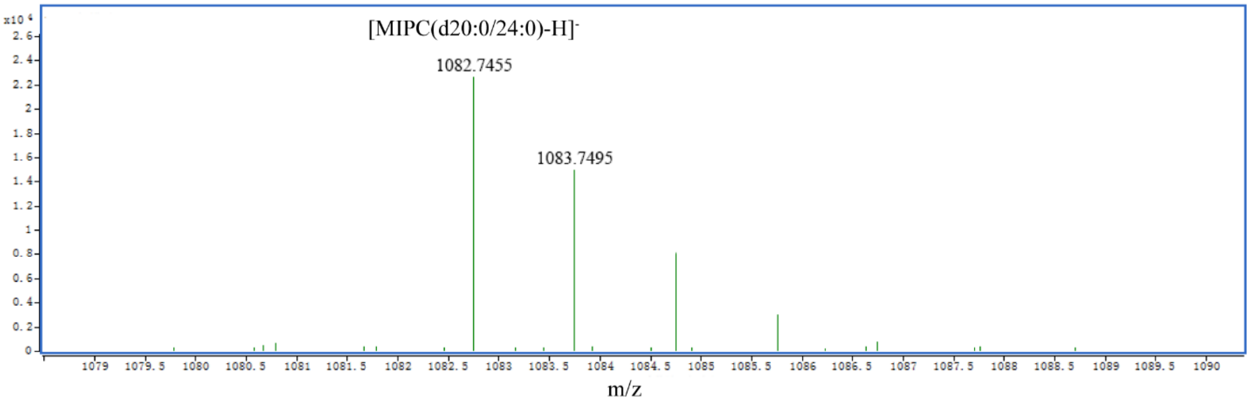
[MIPC(d20:0/24:0)-H]-
1082.7455
1083.7495
x10 4
m/z

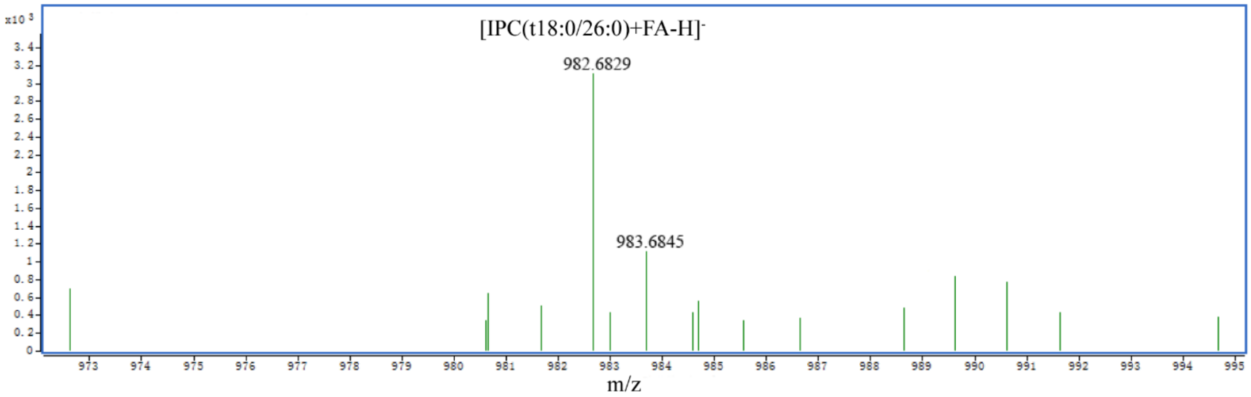
[IPC(t18:0/26:0)+FA-H]-
982.6829
983.6845
x10 3
m/z

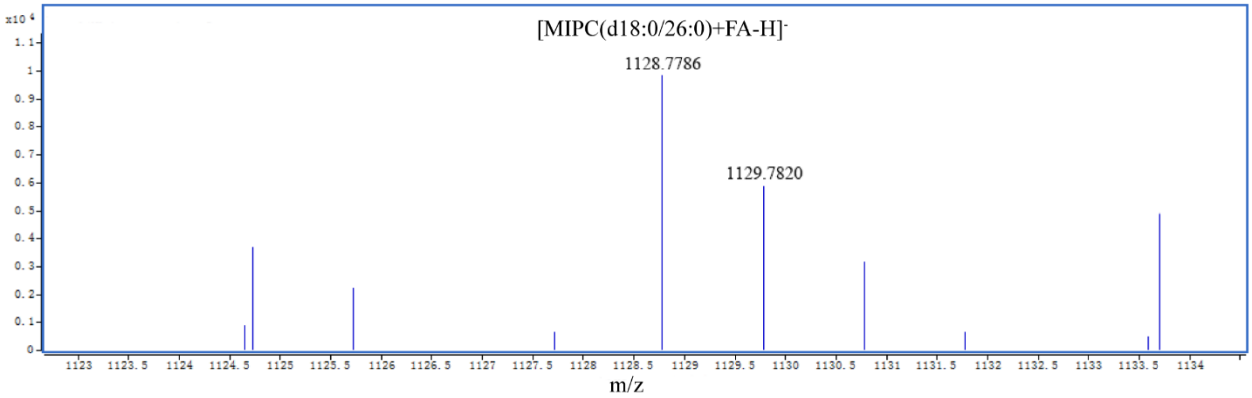
[MIPC(d18:0/26:0)+FA-H]-
1128.7786
1129.7820
x10 4
m/z

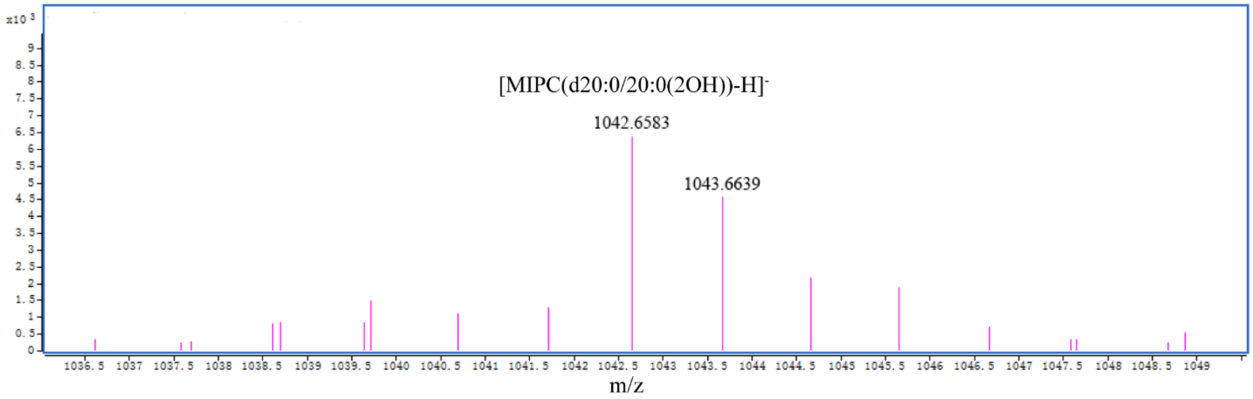
[MIPC(d20:0/20:0(2OH))-H]-
1042.6583
1043.6639
x10 3
m/z

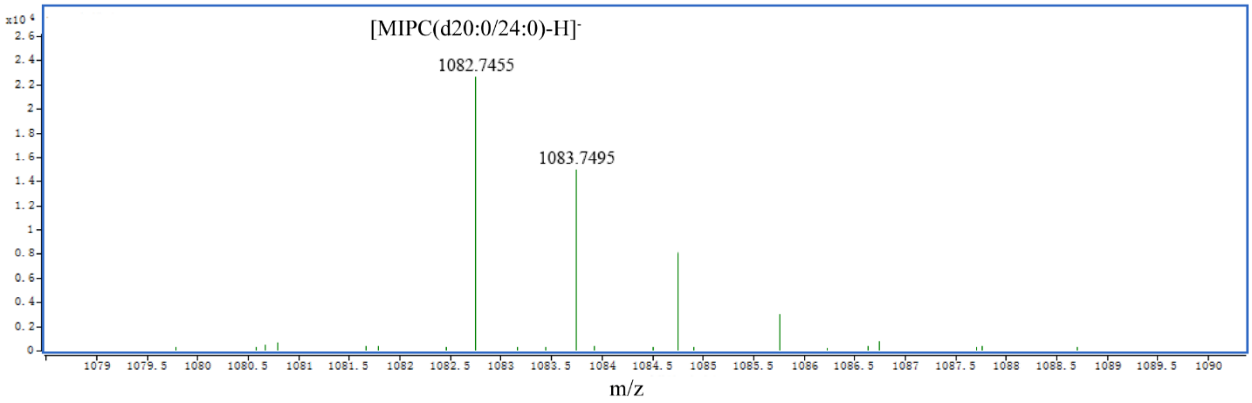
[MIPC(d20:0/24:0)-H]-
1082.7455
1083.7495
x10 4
m/z

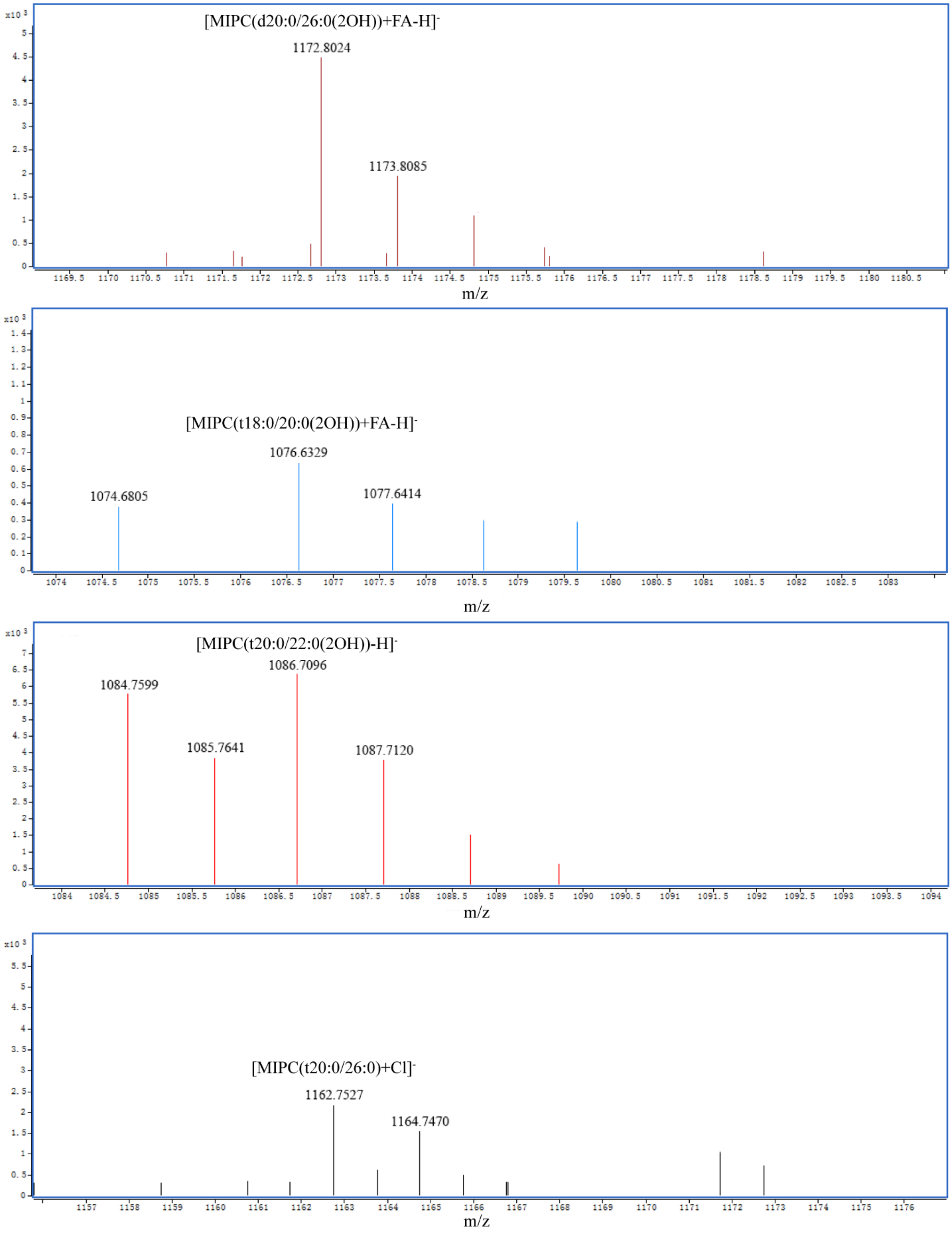
[MIPC(d20:0/26:0(2OH))+FA-H]-
1172.8024
1173.8085
m/z
[MIPC(t18:0/20:0(2OH))+FA-H]-
1074.6805
1076.6329
1077.6414
m/z
[MIPC(t20:0/22:0(2OH))-H]-
1084.7599
1085.7641
1086.7096
1087.7120
m/z
[MIPC(t20:0/26:0)+Cl]-
1162.7527
1164.7470
m/z

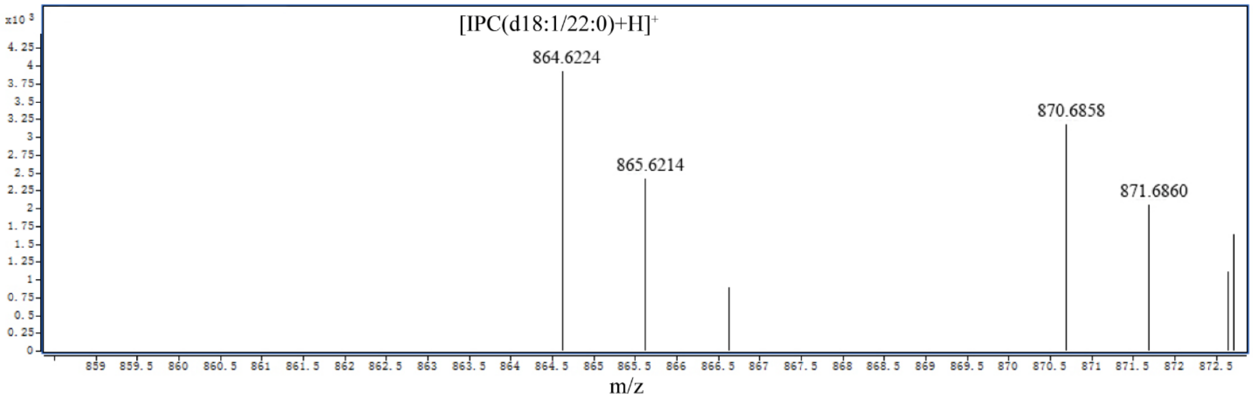
[IPC(d18:1/22:0)+H]+
864.6224
865.6214
870.6858
871.6860
m/z

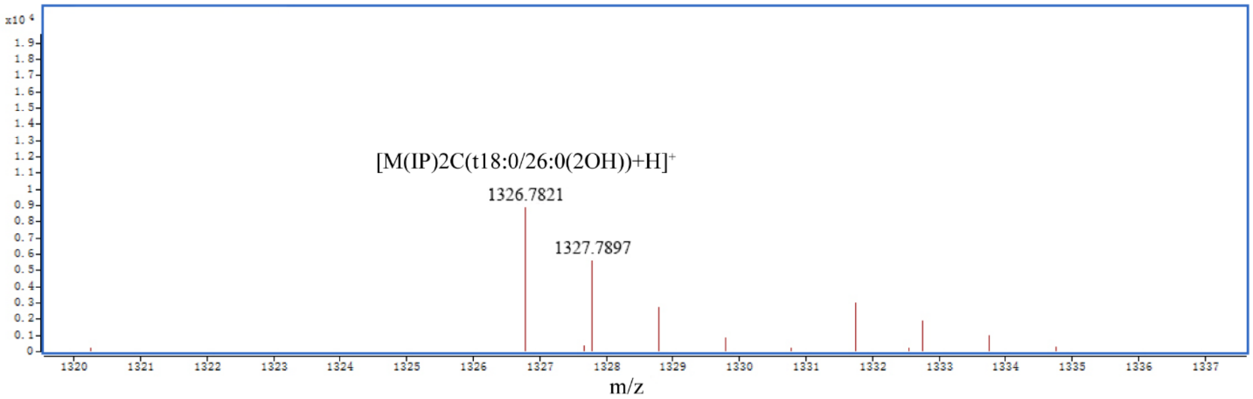
[M(IP)2C(t18:0/26:0(2OH))+H]+
1326.7821
1327.7897
m/z

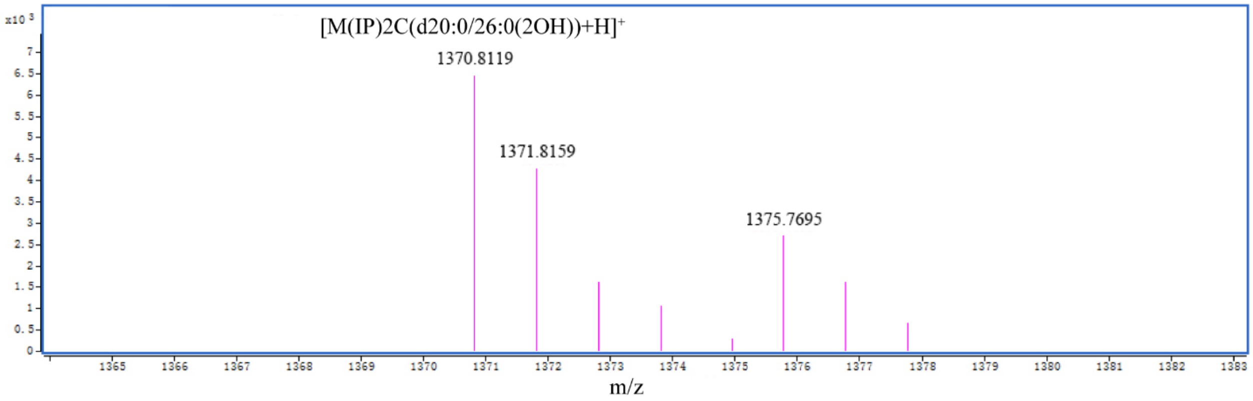
[M(IP)2C(d20:0/26:0(2OH))+H]+
1370.8119
1371.8159
1375.7695
m/z

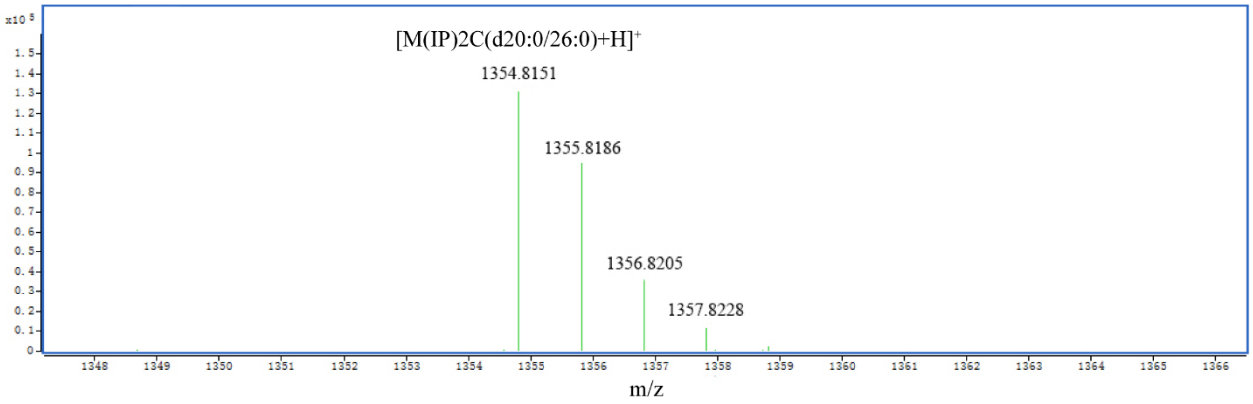
[M(IP)2C(d20:0/26:0)+H]+
1354.8151
1355.8186
1356.8205
1357.8228
m/z

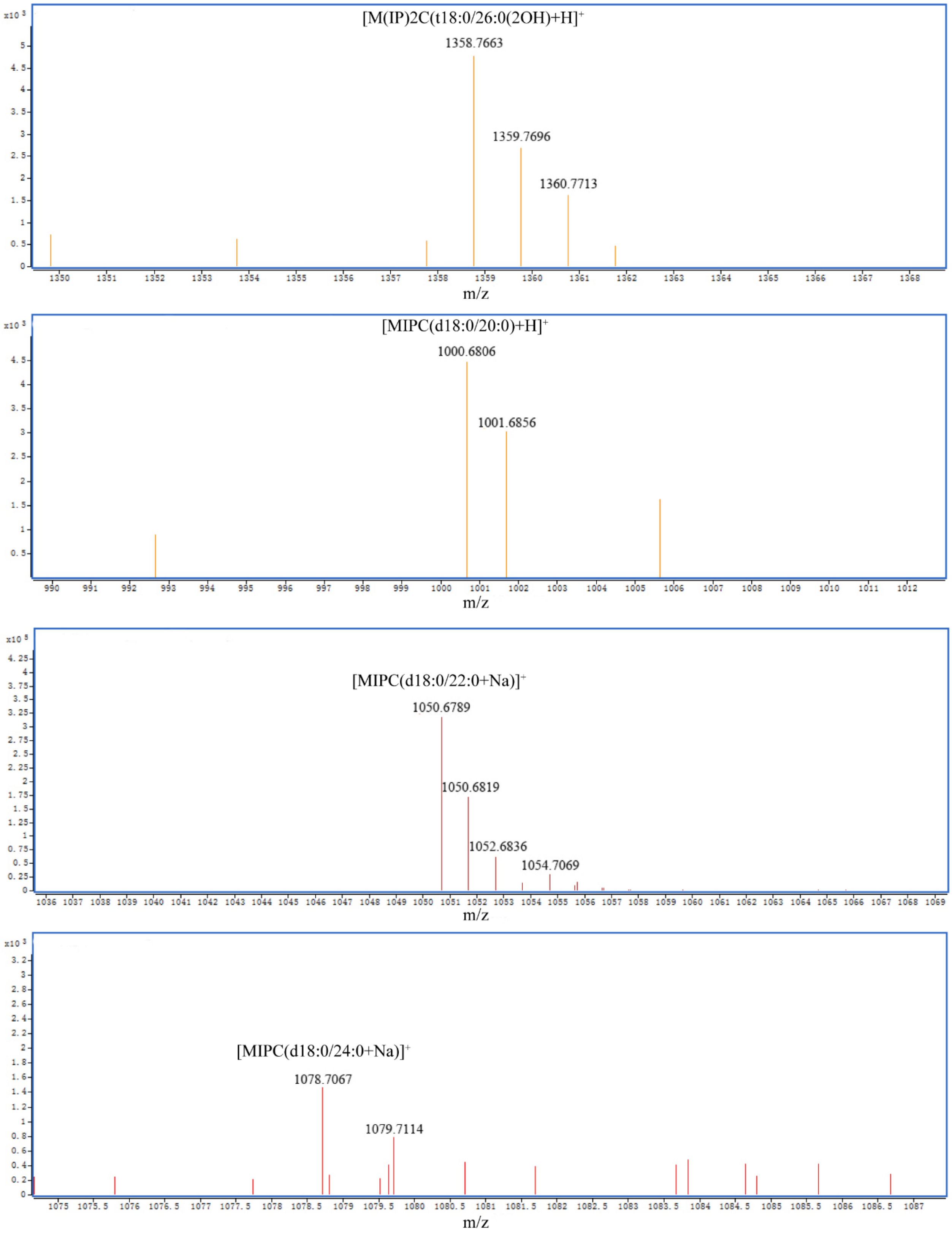
[M(IP)2C(t18:0/26:0(2OH)+H]+
1358.7663
1359.7696
1360.7713
m/z
[MIPC(d18:0/20:0)+H]+
1000.6806
1001.6856
m/z
[MIPC(d18:0/22:0+Na)]+
1050.6789
1050.6819
1052.6836
1054.7069
m/z
[MIPC(d18:0/24:0+Na)]+
1078.7067
1079.7114
m/z

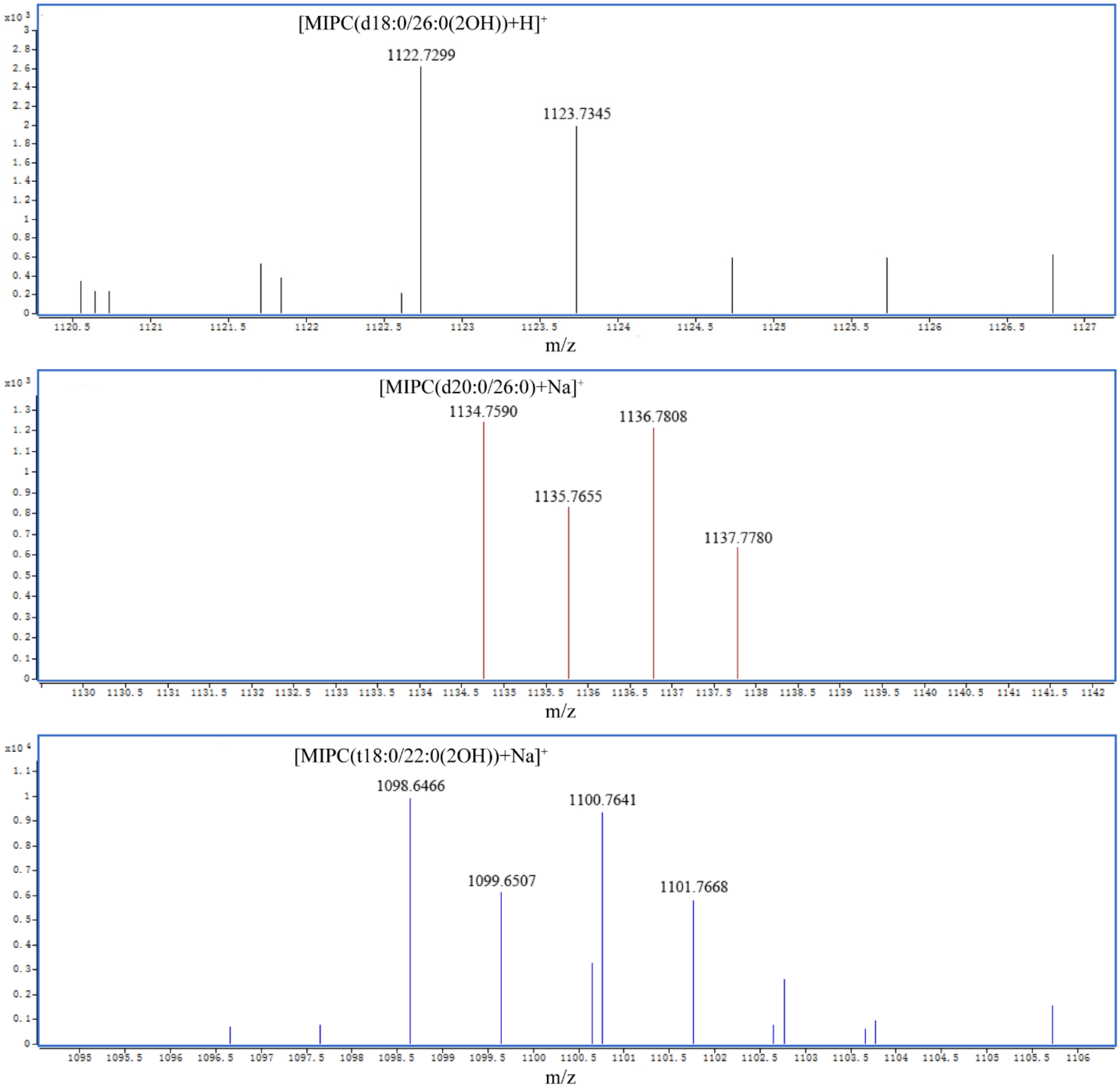


**Fig. S5 Chromatographic analysis of complex sphingolipids.**
